# Reflections on a pandemic and surveillance: Disruption, distractions, and discoveries in the United Arab Emirates

**DOI:** 10.1177/1473325020984157

**Published:** 2021-03

**Authors:** Taghreed M Abu Sarhan

**Affiliations:** Social Wellbeing Department, College of Humanities and Social Sciences, United Arab Emirates University, Al Ain, UAE

**Keywords:** Technology, resilience, human rights, COVID-19, surveillance, human dignity

## Abstract

Reflecting on experiences in the United Arab Emirates, one professor’s thoughts on surveillance and human dignity is explored. Different forms of surveillance are touched upon, including the use of ankle bracelets to track those who are infected with COVID-19. Human rights are touched upon offering observations and closing with the fact that the number of deaths, in the UAE, have been exceptionally low.

On March 5, 2020 my employer, the flagship university in the United Arab Emirates (UAE), announced a cessation of in-person learning. Like many educators around the world, we were asked to quickly stand up online learning. For us, in social work, we had the inevitable challenges of technology combined with the need to teach a practice-based curriculum. There were important lessons in this experience and I continue to reflect on this time of rapid change. While this topic of educating social workers in the online environment is interesting, I am rather focusing on a lesser discussed topic amongst social work educators. And, that is the ability for our government institutions to take a strong stance on social and physical distancing, aided by technological advances. Specifically, in the United Arab Emirates we experienced some of the most aggressive digital and other measures—many based on technology—to stop the spread of COVID-19.

While social work faculty in the West are often oriented to conceptions of *liberty* that focus on individual freedoms, in the Middle East our collective vision of family and community responsibility offers a different paradigm of ‘freedom.’ As I write this reflection as a Jordanian woman, I bring that lens to this reflection and I write from a strengths and resilience perspective related to the United Arab Emirates response to COVID. I present my reflection with the recognition that readers in the West may find my observations to be counter to social work values. However, I also recognized that with the rapid rise of social work education in the Arab-Muslim world there is a need to add to the discourse about COVID from our cultural perspective. Furthermore, because I focus on human rights as a focal point of my scholarship, I will approach this reflection with a framing of the major ideas with a *human dignity* overlay. Because this reflective piece is relatively short, greater expansion on human rights is not possible.

The idea of surveillance has different meanings depending on the usage of the word. In public health, surveillance is based on epidemiology and the capacity of a community or society to count the morbidity and mortality of a disease. Being able to surveil a community has a long history now, these ideas date well over a hundred years now when boys who were chimney sweeps in London were found to have testicular cancer in significant numbers. These cases were so remarkable that, in time, this form of cancer was eventually recognized as a risk factor for the occupation for chimney sweeps. Of course, surveillance with epidemiological data gathering was rudimentary at this stage. However, from a public health perspective, we now have a very sophisticated and much needed system to identify morbidity and mortality. For COVID-19, surveillance is a daily reality globally as the World Health Organization as well as governments around the world make decisions in the best interests of their general population.

As the UAE had one of the first responses to closing universities—very, very early—we lived through the earliest transformations to online learning as a result of infectious disease. For us, as faculty, the transition was quick and painful, at times. However, I was most concerned about my students who really needed a classroom presence to keep them motivated. It became more and more clear to me as I was able to observe how many students were actually participating in online learning. That is, logging into the online platform, participating in discussion groups and responding to blogs and so forth. Quite obviously, in these early days, we were all just surviving and finishing the semester which happened to overlap with the beginning of Ramadan. We entered the season of prayer and gratitude with the thankfulness that we were all alive and able to fast after such a challenging semester.

However, as we move forward, I am all too aware of how surveillance can now be used to observe both student behaviors (amount of usage of online resources, etc.) as well as faculty time spent on teaching and learning activities. The ability to measure productivity with surveillance will be an interesting path to observe as we adjust to the new realities of COVID-19. Obviously, as educators, we will have to ponder the inevitable surveillance challenges in this area as we continue with online education.

Another element of surveillance that we all experienced in the UAE was the ability of the government to track the movements of those infected with COVID-19. If and when an individual tested positive for the virus, they were fitted with an ankle tracking bracelet that is the same used for criminals on house arrest. However, in this case the bracelet was to track the movements of those ordered into quarantine due to illness. Then, the government arranged for meals to be delivered to those who needed food service as a result of the quarantine restrictions. In the West, such a policy position may look punitive and as a result of the lack of government trust in its people. However, in a collectivist society with great access to technology, we understood the policy position and recognized that ankle bracelets and such monitoring was for the greater good. Furthermore, the government made many accommodations, including rapid and frequent testing, to aid those infected with COVID-19 in their recovery and eventual exit from quarantine status. Once you test negative, the ankle bracelet is removed.

Other surveillance is related to the movement of peoples between the different states of the UAE. Restricted movements were monitored by police that checked relevant papers at barricades throughout the country. Restricting movement, in such a small country with less than 10,000,000 people was quite obvious. However, again we understood the aggressive policy position and the need for police to act as enforcement agents. In realty, in the Middle East, the police are involved in all aspects of society and especially responses to family needs and wellbeing. This is evidenced by the family support centers that are directly connected to police functioning; often they are even co-located in the community so that families with problems can immediately be referred to social work services in adjoining buildings. So, police enforcement of social protection measures is not new to us, as people of the Middle East. We recognize the essential role of police in public safety and societal functioning.

These are just a few of the surveillance steps made by the government. We have been constantly updated as a population with text messages to inform of curfew times and related activities such as disinfecting the metro system in Dubai and later all cities and towns of the country. Over time we have been informed frequently related to updates and new policies and procedures aimed at safety. For me and many of my friends and colleagues, we had a sense of relief to be given frequent information about the *how, what,* and *when* of the public health efforts.

As professors, we also were comforted by the updates that we have received from the university. We have a sense of transparency as our employer provides, on a regular basis, precautions that are necessary to access our offices, libraries and other campus facilities. Also, because the vast majority of faculty are foreigners rather than citizens—travel to other places in the world to be reunited with family is a common concern. With sensitivity to this reality, the university has been dedicated to the best information possible and explaining travel procedures inside and outside of the country.

From a human rights perspective, I am reminded of the ideas set forth by philosophers and their interpretation of *dignity and worth of the person***.** The “Preamble” to the United Nations Universal Declaration of Human Rights, for example, invokes considerations of human dignity. The term dignity is used frequently in moral discourse (Pullman, 2002). Human dignity and worth of person are uniquely valuable and therefore ought to be given the highest respect and care (Andorno, 2010).

Since 1948, with the passage of the Universal Declaration of Human Rights, international human rights law has been grounded on the assumption that human beings need to enjoy their equal basic rights with which they were born. Human dignity and worth of the person have been recognized by the international community as the foundation upon which human rights are based, Philosophers assume that “Human dignity holds also a prominent position in the intergovernmental instruments relating to biomedicine that have been adopted since the end of the 1990s by bodies such as UNESCO, the United Nations, and, at the European level, the Council of Europe” (Andorno, 2010: 1)

## Conclusion

As a faculty member of social work and after having worked a 17-year career in family protection as a police officer in Jordan, I welcome this opportunity to *reflect* upon my experiences in the UAE. I am quite grateful that my family is safe here and I do worry about my older mother and greater family in Jordan. However, here in the UAE, I know that the government is using technology to keep us all safe. Their diligence, as a government, is one that will likely be misunderstood in the West. This is especially true for the use of ankle bracelets that monitor movement of people testing positive for COVID-19. This may seem like a form of criminalization, but we do not see these measures negatively. We recognize the importance of safety for all. Fundamentally, the conceptions of freedom are different in the Middle East. Freedom from death and disease is the ultimate goal in this time of health emergency. In the end, one can say that the UAE has been very successful as the infection and disease rate is quite low. Some 55,255 cases of infection have reported with only 338 deaths documented as of July 12, 2020.
